# Intracavitary hydatid cyst of the left ventricle in child: an emergency surgery case report

**DOI:** 10.11604/pamj.2014.19.401.5770

**Published:** 2014-12-30

**Authors:** Hicham El Malki, Abdelkhalek Chetoui, Hicham Fenane, Hicham Benyoussef, Jaafar Rhissassi, Rochde Sayah, Mohamed Laaroussi

**Affiliations:** 1 Department of Cardiovascular Surgery A, Ibn Sina Hospital, Rabat, Morocco; 2 Department of Thoracic Surgery, Ibn Sina Hospital, Rabat, Morocco

**Keywords:** Hydatid cyst, left ventricle, cystectomy

## Abstract

Hydatid cysts of the heart are very rare in child. In this report we describe an interesting and unique case of an 8-year-old boy with a large cardiac intracavitary hydatid cyst filling the left ventricle. Echocardiography, computerized tomography, magnetic resonance imaging and serologic test were necessary for the diagnosis. Once assessing the diagnosis, an emergency open heart surgery was necessary to prevent the complications. Surgery associated to medical treatment provides good results as demonstrated in this case report.

## Introduction

Echinococcosis is a serious health issue in some geographical regions of the world [1]. Thoracic involvement is dominated by the lung. Hydatid cysts (HC) of the heart are rare. The clinical diversity of cardiac echinococcosis explains the several surgical treatment options depending on the cyst location and its complications [2]. In this report we describe an interesting and unique case of a large cardiac intracavitary hydatid cyst filling the left ventricle in a child. Management strategies are discussed and our treatment approach is presented.

## Patient and observation

**Figure 1:**
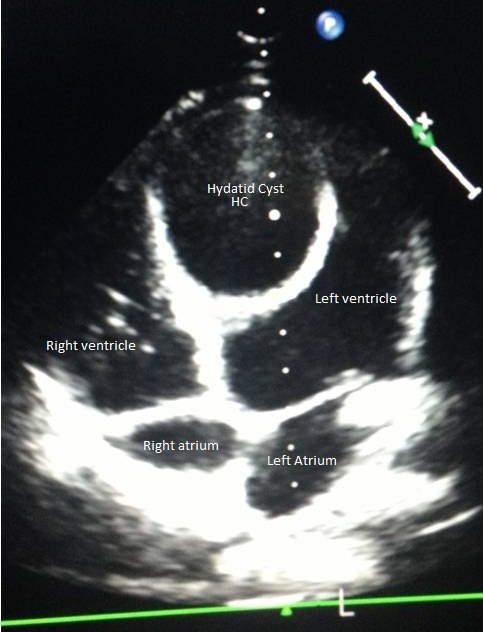
TTE: echocardiographic appearance of left ventricular hydatid cyst

**Figure 2:**
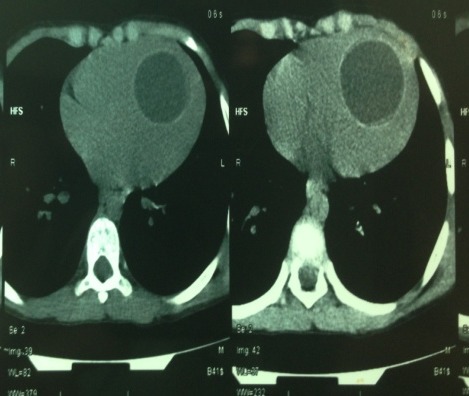
CT Scan: a hypodense round liquid formation in the left ventricle

An 8-year-old boy living in rural areas was admitted to the pediatric department for lung infection for which a chest X-ray showed a cardiomegaly. An echocardiographic examination was then performed and the diagnosis of a cardiac hydatid cyst was established. After beginning a medical treatment of the pulmonary infection, the patient was referred to our clinic for the treatment of his cardiac HC. He had no angina, dyspnea, nor heart failure and had no cardiovascular disease history. The family history was unremarkable. The physical examination and ECG were unremarkable. Laboratory investigations were normal. Serology for ecchinococcus antibodies was negative. The chest X-ray revealed an increased cardiothoracic index, while the pulmonary parenchyma was normal. Transthoracic echocardiogram showed a large hypoechoic cystic mass, measuring 41 x 55 mm (area = 19 cm^2^) with liquid content in the left ventricle (LV) (Figure 1). This unilocular mass was located in the left ventricle lumen and attached to the interventricular septum. Left ventricular function was well preserved. A contrast-enhanced CT scan showed a low-attenuation mass in the left ventricle (Figure 2), a finding that is consistent with type I unilocular HC. Cardiac-gated magnetic resonance imaging confirmed the presence of the HC in the LV and described its relationship with cardiac chambers. On searching for other associated lesions by brain and abdominal CT: Other systemic organs were not involved.

**Figure 3:**
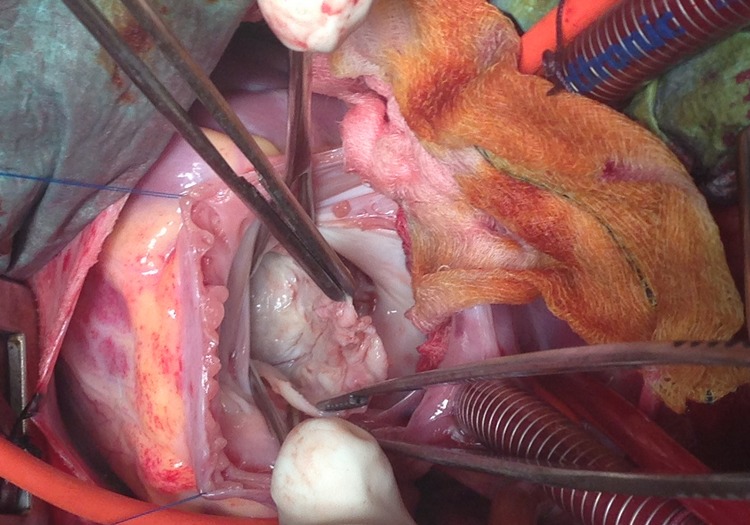
Surgical view after cardiac arrest and cardiac incisions showing the white mass (hydatid cyst) through the mitral valve

**Figure 4:**
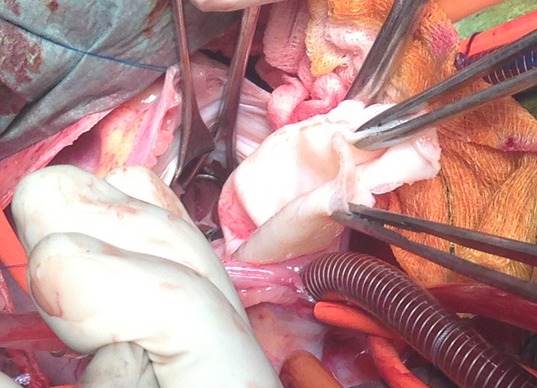
Surgical view while removing the intracavitary left ventricular hydatid cyst

**Figure 5:**
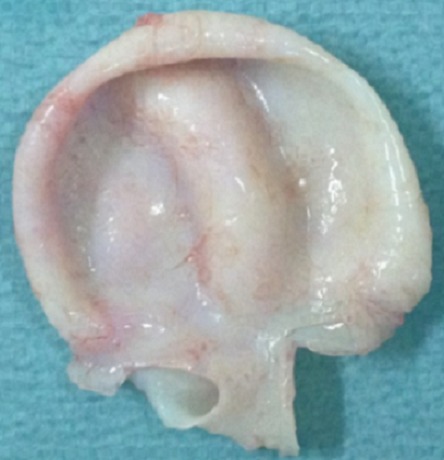
Left ventricular hydatid cyst once removed

The inherent risks of such a large primitive intracavitary cardiac hydatid cyst are rupture, embolism, anaphylaxis, ventricular dysfunction or heart failure. Performing a curative surgery in emergency is the only way to prevent all these complications. The child underwent, through a median sternotomy, an open heart surgery using routine cardiopulmonary bypass between the ascending aorta and the two laced vena cava veins (aortic and pulmonary artery cross clamping). After right atriotomy and through the atrial septum and the mitral valve, a 5 cm white mass was found on the left midventricular part of the muscular interventricular septum (Figure 3). Protection was done with pieces of gauze soaked with hypertonic saline. Polyvinylpyrolidone iodine solution was injected into cysts to inactivate the scolexes. To prevent contamination of the surrounding area, cyst fluid was aspirated through a needle puncture and then cyst was carefully opened. The germinative membrane and daughter cysts were removed as a whole (Figure 4). The residual cavity layered by fibrous wall was cleansed by wiping with polyvinylpyrrolidone solution and was not closed to avoid conduction disturbances and complete atrioventricular block. Histopathologic examination of surgical specimen (Figure 5) confirmed the diagnosis of hydatid cyst.

The immediate postoperative period was uneventful with a short stay (48 hours) in the intensive care unit (ICU). ECG still unchangeable. The patient was discharged from the hospital on the 8th postoperative day with albendazole (10 mg/kg daily) treatment for 6 months to prevent recurrence. At the 18 month follow up there was no recurrence of hydatid disease.

## Discussion

Cardiac Hydatidosis are rare: they account for only 0.5 to 2% of hydatid locations [1]. There does not appear to be any age limit at presentation; it may manifest even in early childhood. However, cardiac hydatid cyst in child still unusual through literature and the mean age in all the series or cases is above 30 year-old [1, 3–6]. The impingement mostly occurs by contiguity from a mediastinal or pericardial HC. Primitive implantation, as described in this case, is very rare and is even rarer through blood. The parasite can be conveyed directly by the vasa vasorum into the cardiac wall or hung by preexisting intimal lesions or trapped in small aneurysms during its transit in the mediastinal great vessels [1]. The evolution is marked by gradual appearance of inflammatory pericystic phenomenon which weakens the cardiac wall. The left ventricle is the most common site (55%-60%) due to the thickness of myocardium and less vascular character [1], followed by the right ventricle (15%), interventricular septum (9%), left atrium (8%), right atrium (4%) and interatrial septum (2%) [1]. Cardiac hydatidosis may lead to valve destruction and valve replacement may be necessary, or it may simulate valvular lesions. Conduction disturbances and complete atrioventricular block may be caused by cysts close to the interventricular septum [2, 3].

Clinical presentation differs according to the number, size, localization, and complication of the cysts, which also makes early diagnosis difficult [7]. Echocardiography, computerized tomography, and magnetic resonance imaging are valuable diagnostic tools [5]. A correct treatment of this rare disease is very important. Hydatid cyst fluid is a very potent anaphylactic substance [4]. If a hydatid cyst ruptures into the cavity, the cyst fluid may cause anaphylaxis and the cyst membrane may cause an embolus [5]. So treatment is essentially surgical. It involves identifying and eliminating the seed source. To avoid complications, open heart surgery in emergency with cardiopulmonary bypass is preferred as it allows systemic arterial clamping. This prevents migration of scolexes and minimizes the risk of dissemination in case of cyst break [1, 4]. The procedure involves puncturing and sterilizing the HC, before removing it and then performing a cystectomy. It is important to avoid as much as possible decaying incisions such as left ventriculotomy in order to minimize post-operative left ventricular failure risk [1].

Pre and post-operative medical adjuvant therapy with albendazole helps to limit the risk of dissemination by accidental hydatid release [1, 4, 6]. The postoperative period is generally uneventful. However clinical, serological and radiologic (TTE, CT, MRI) follow-up is required to check for pseudo-aneurysm formation and any recurrence.

## Conclusion

Hydatid cysts of the heart in child are rare and serious. Late diagnosis of an intracavitary HC of the left ventricle is responsible for poor prognosis linked to the rupture risk, the haematogenous dissemination risk and the left or right ventricular dysfunction risk. An emergency open heart surgery is necessary once intracavitary left ventricle HC is diagnosed. Surgery associated to medical treatment provides good results as demonstrated in this case report.
